# Continuing difficulties in interpreting CNV data: lessons from a genome-wide CNV association study of Australian HNPCC/lynch syndrome patients

**DOI:** 10.1186/1755-8794-6-10

**Published:** 2013-03-26

**Authors:** Bente A Talseth-Palmer, Elizabeth G Holliday, Tiffany-Jane Evans, Mark McEvoy, John Attia, Desma M Grice, Amy L Masson, Cliff Meldrum, Allan Spigelman, Rodney J Scott

**Affiliations:** 1 School of Biomedical Sciences and Pharmacy, University of Newcastle, Newcastle, NSW, Australia; 2 Hunter Medical Research Institute, John Hunter Hospital, Newcastle, NSW, Australia; 3 School of Medicine and Public Health, University of Newcastle, Newcastle, NSW, Australia; 4 Food and Nutritional Sciences, Preventative Health Flagship & CSIRO, North Ryde, NSW, Australia; 5 Hunter Area Pathology Service, Hunter New England Area Health, New Lambton Heights, NSW, Australia; 6 University of NSW, St Vincent’s Hospital Clinical School, Sydney, Australia; 7 Hunter New England Family Cancer Service, Newcastle, NSW, Australia

**Keywords:** HNPCC, Lynch syndrome, SNP arrays, CNVs, CNV burden

## Abstract

**Background:**

Hereditary non-polyposis colorectal cancer (HNPCC)/Lynch syndrome (LS) is a cancer syndrome characterised by early-onset epithelial cancers, especially colorectal cancer (CRC) and endometrial cancer. The aim of the current study was to use SNP-array technology to identify genomic aberrations which could contribute to the increased risk of cancer in HNPCC/LS patients.

**Methods:**

Individuals diagnosed with HNPCC/LS (100) and healthy controls (384) were genotyped using the Illumina Human610-Quad SNP-arrays. Copy number variation (CNV) calling and association analyses were performed using Nexus software, with significant results validated using QuantiSNP. TaqMan Copy-Number assays were used for verification of CNVs showing significant association with HNPCC/LS identified by both software programs.

**Results:**

We detected copy number (CN) gains associated with HNPCC/LS status on chromosome 7q11.21 (28% cases and 0% controls, Nexus; *p* = 3.60E-20 and QuantiSNP; *p* < 1.00E-16) and 16p11.2 (46% in cases, while a CN loss was observed in 23% of controls, Nexus; *p* = 4.93E-21 and QuantiSNP; *p* = 5.00E-06) via *in silico* analyses. TaqMan Copy-Number assay was used for validation of CNVs showing significant association with HNPCC/LS. In addition, CNV burden (total CNV length, average CNV length and number of observed CNV events) was significantly greater in cases compared to controls.

**Conclusion:**

A greater CNV burden was identified in HNPCC/LS cases compared to controls supporting the notion of higher genomic instability in these patients. One intergenic locus on chromosome 7q11.21 is possibly associated with HNPCC/LS and deserves further investigation. The results from this study highlight the complexities of fluorescent based CNV analyses. The inefficiency of both CNV detection methods to reproducibly detect observed CNVs demonstrates the need for sequence data to be considered alongside intensity data to avoid false positive results.

## Background

Genetic variation explains a significant proportion of susceptibility to common disease [1-5]. Copy number variations (CNVs) are quantitative structural genetic variations affecting the number of copies of a particular genomic region (deletions or duplications of DNA segments) ranging from a kilobase to several megabases in size and covering 12% of the human genome [6,7]. Different populations share a large percentage of CNV regions and the closer the relationship, the greater the sharing [8], e.g. populations from different continents share ~40% of CNVs, while populations within the same continent share ~50%. The widespread distribution of CNVs across the genome suggests they can account for a proportion of population variation in common disease status. Mechanisms by which CNVs may influence disease risk include; impacting gene expression due to interruption of coding sequences, gene dosage or on neighbouring gene regulation [9-14]. Evidence reported in the literature clearly shows that CNVs play an important role in disease development and phenotype expression [14-18]. Chromosomal deletions have been found more prevalent than duplications in case–control studies [19,20] and interestingly, microRNAs (miRNAs) and miRNA-binding sites are concentrated in CNV regions [21]. It has been suggested that the development of a disease phenotype may not depend upon a single CNV but rather a combination of various CNVs and other genetic variations (e.g. single nucleotide polymorphisms (SNPs)) [14]. Indeed, an increased global CNV burden has been observed in schizophrenia, autism and short stature which has been used as model for polygenic traits that are highly heritable [20,22-24]. Nevertheless, studies of CNV burden have also yielded inconclusive results [25].

SNP genotyping platforms have been used for genome-wide association studies (GWASs) to identify novel cancer susceptibility loci [26,27], resulting in the discovery of many novel low-penetrance colorectal cancer (CRC) loci [28-32]. The ability to perform genome-wide studies of large CNVs has been facilitated by advances in array comparative genomic hybridisation (array CGH) and the development of CNV calling algorithms based on high throughput SNP genotype data [33,34]. Several new candidate genes potentially predisposing to early onset CRC have been identified utilising arrayCGH [35]. In light of these findings we used a high density SNP genotyping array, combined with *in silico* CNV calling to search for CNVs potentially involved in hereditary non-polyposis colorectal cancer (HNPCC)/Lynch syndrome (LS). HNPCC/LS is an autosomal dominantly inherited cancer predisposition associated with mutations in DNA mismatch repair (MMR) genes or genes affecting expression of MMR genes [36-41]. MMR proteins confer several genetic stabilisation functions; they correct DNA biosynthesis errors, ensure the fidelity of genetic recombination and participate in the earliest steps of cellular checkpoint control and apoptotic responses [42]. MMR gene defects increase the risk of malignant transformation of cells, ultimately resulting in the disruption of one or several genes associated with epithelial integrity. Current classification schemes differentiate between LS patients harbouring mutations in MMR genes and HNPCC patients who adhere to the Amsterdam Criteria [43] or Bethesda guidelines [44] where a pathogenic mutation in MMR genes has not been detected with existing screening strategies. HNPCC is also referred to as familial colorectal cancer-type X [45]. The population frequency of LS has been estimated at approximately 1 in 3000 individuals [46,47]. By 70 years of age, 45% of men and 33% of women diagnosed with HNPCC/LS will develop CRC and 15% of women will develop endometrial cancer [48,49]. Given that mutations in DNA mismatch repair genes are generally not considered to display distinct genotype-phenotype correlations and are only identified in ~50% of individuals with a clinical diagnosis of HNPCC, it has become apparent that other genetic factors are likely to influence disease development.

The aims of the current study were to search for genomic regions that might contribute to the development of CRC in HNPCC/LS families by identifying CNVs which differentiate HNPCC/LS cases from healthy controls and to ascertain the extent of genomic CNV burden between cases and controls and in the context of potential MMR deficiency. We have identified one locus in an intergenic region on chromosome 7q11.21 possibly associated with disease risk in patients diagnosed with HNPCC/LS and observed a greater CNV burden in cases compared to controls. The results from this study highlight the complexities of fluorescent based CNV analyses.

## Methods

### Samples

The sampling frame for this study was 833 LS/HNPCC families tested at Hunter Area Pathology Service between the years of 1997 and 2010. All patients included in the study gave informed consent for their de-identified DNA and clinical records to be used for future research related to their condition. Ethics approval was obtained from the Hunter New England Human Research Ethics Committee and the University of Newcastle’s Human Research Ethics Committee. Each participant had previously contributed blood from which DNA was extracted using the salt precipitation method [50]. For the current study, from the set of available families we selected 100 unrelated LS/HNPCC cases (see Table 1) and 384 healthy controls from the Hunter Community Study [51].

**Table 1:** Illustration of samples used in the study

	**Cases**	**Diagnosed with extra colonic cancer**	**Diagnosed with a second CRC**
LS cases (mutation positive)	64*	23 (36%)	14 (22%)
HNPCC cases (mutation negative)	36**	10 (28%)	0
Total	100	33 (33%)	14 (14%)

* All but 3 adhered to the Amsterdam II criteria or the Bethesda guidelines.

** All but 1 adhered to the Amsterdam II criteria or the Bethesda guidelines.

### Genotyping and identification of Copy Number Variation (CNV)

All samples were genotyped using the Illumina Human 610-Quad BeadChip (Illumina Inc., San Diego, CA, USA) which contains 620,901 markers. In addition to evenly spaced and comprehensive tag SNPs the Human610-Quad BeadChip includes ~60,000 CNV-targeted markers in regions containing known CNVs. Median spacing between markers is 2.7 kb (mean 4.7 kb). The BeadChips were processed by the same technician over a three months period; the samples were not randomized as the control cohort used for this study was part of a larger longitudinal study of health and aging in healthy adults [51]. BeadChip data was processed using GenomeStudioV2010.1 (Illumina Inc.) according to the manufacturer’s description. Primary data analyses, including raw data normalisation, clustering and genotype calling were performed using algorithms in the genotyping (GT) Module. The software derives, for each sample, log R ratios (LRR) and B allele frequencies (BAF) for each probe on the 610-Quad array; the LRR reflects relative probe fluorescence intensity, which varies with the discrete number of copies of probe-specific DNA present within an individual’s genome.

The LRR and BAF values were used as input for Nexus Copy Number Software, Version 5 (BioDiscovery, El Segundo, CA, USA). A copy number state of 2 per individual is considered normal (one copy per chromosome); lower values reflect copy number loss and higher values a copy number gain. Nexus, used for CNV calling and association analyses, offers several algorithms for CNV detection; the SNPRank segmentation algorithm was used based on a previous report demonstrating its low type I error rate and high power compared with a range of available algorithms [34]. A significance threshold of 0.01 was required for declaring the presence of a segment; a threshold empirically identified as the value which minimised the normalised singleton ratio (NSR) parameter in a training set of ten randomly chosen samples from our sample cohort. NSR has previously been demonstrated as a useful optimisation parameter for CNV detection and is calculated as the proportion of unique CNV SNPs found in only 1 sample divided by the average number of CNV SNPs called per sample [34]. The following analyses settings were applied in Nexus to define a CNV: A minimum of 5 probes per segment (increased from default 3), high gain = 0.6 LRR (default), gain = 0.18 LRR (default), loss = −0.18 LRR (default) and big loss = −1.0 LRR (default). For the association testing the p-value threshold was also set to 0.01 and the differential threshold was set at default 25%.

To minimise the impact of type I error (resulting from incorrectly called CNVs) upon our conclusions, all CNV regions demonstrating significant association using Nexus (see below for statistical methods) were independently tested for validity using QuantiSNP software Version 2.3 Beta (Wellcome Trust Centre for Human Genetics) [52]. Both programs infer CNV states from array data based on LRR and BAF; the difference between the two methods is the iterative mathematical methods utilised: QuantiSNP uses an objective Bayes hidden-Markov model, while Nexus SNPRank uses a segmentation algorithm (a variation of the circular binary segmentation) that recursively divides chromosomes into segments of common intensity distribution. The default program settings were used in QuantiSNP for defining copy number states: EM-iters – the number of iterations used in training the model on the experimental data; L-setting – expected typical number of base pairs in a CNV-region (smoothing factor); and the maxcopy setting – maximal CN state value in the Markov model. GC-correction is applied by default to smooth out genomic waving. Association analyses was performed only for CNVs called with high confidence (maximum log Bayes Factor >10) containing a minimum of 5 probes (changed from default 10).

As a final step to confirm the validity of events with significant frequency differences, log R ratio plots were visually inspected to ensure that called CN gains and losses were visually evident and not simply artefacts of the calling algorithm. Human genome build 18 (36.3) has been used for assigning chromosome positions throughout the manuscript.

### Statistical analyses

Significance testing for group differences in individual CNV frequencies was performed utilising Nexus Protocol 5 [53] for Nexus output, and t-test implemented in Stata (version 10, StataCorp, College Station, TX) for QuantiSNP results, where the predictor variable was the quantitative copy number at each locus. To control the family-wise type I error rate (FWER) across multiple tests, a significance threshold of p < 3.6E-06 was used for significance tests of individual CNV events; a threshold derived by dividing the desired FWER of 0.05 by the total number of non-overlapping CNVs tested for significance. We note that this Bonferoni threshold is likely conservative, owing to probable linkage disequilibrium (correlation) between some adjacent, non-overlapping CNVs. Statistical comparisons were performed only for CNV events observed on autosomes, due to the complexity of analysing the X and Y chromosomes.

For each individual, we also defined several measures of autosomal genomic CNV burden and compared mean values between phenotypic groups. CNV burden for each individual was defined in three distinct ways; 1) the total length of genomic DNA involved in identified CNV events; 2) average length of CNVs; and 3) total number of CNVs. Comparison of CNV burden between groups was conducted using t-tests in Stata.

All results reported as statistically significant have reached our pre-specified, adjusted significance threshold using both Nexus and QuantiSNP results and also shown a consistent direction of effect (frequency difference) using both algorithms.

### Validation of significant CNVs

TaqMan® Copy Number Assays (Applied Biosystems, Carlsbad, CA, USA) were used to validate copy number state for CNVs showing significant case–control frequency differences via *in silico* analyses. Because a Custom TaqMan® CN assay could not be designed for the CN gain region of significance on chromosome 7q11.21 (61,682,801-61,827,108), a neighbouring down-stream sequence between SNPs rs8188515 (61,789,558) and rs4718336 (61,990,710) was submitted for Custom assay design [NT_007933.15] – with forward and reverse primers starting at position 61,860,925 and 61,861,034, respectively (for primer and probe sequences, see Table 2). Most of the samples harbouring the CN gain at 7q11.21 had a longer gain than the associated region, which overlapped the assay design region. Therefore this substitution was acceptable for validation. For the chromosome 16p11.2 CN gain (32,411,929-32,504,942) the sequence between rs28778587 (32,419,415) and rs4368167 (32,489,319) was used for assay design [NT_010393.16] – with forward and reverse primers starting at position 32,453,256 and 32,453,355, respectively (for primer and probe sequences, see Table 2). Both sequences were run on SNPmasker [54] before design submission to eliminate allele specific amplification as a result of SNPs in the primer regions (as required by the manufacturing company).

**Table 2:** Primer and probe sequences for TaqMan® Custom CN assays

**CNVchr7_CCHSNR9**	**Sequence**	**hg18**	**hg19**
Forward primer	TTCTAGTTTTTAGCAGAAAGTATTTCCTTCTTCA	61,860,925	62,223,490
Reverse primer	TTTCATTCAGCTGTTTGGAAACACTATTTT	61,861,034	62,223,599
FAM-dye labelled probe	CATAGGCCTCAATGCGCTCCCAA	61,860,960	62,223,525
**CNVchr16_CCD1S9P**	**Sequence**	**hg18**	**hg19**
Forward primer	CTCCCAAATGTCCATTCACCAAAT	32,453,256	32,545,755
Reverse primer	TTTCTTATGTGTGCATTATTCTCACAGA	32,453,355	32,545,854
FAM-dye labelled probe	**ACCTTTCCTTTGATTCAGCAGTTTT**	32,453,299	32,545,798

The Custom TaqMan® CN Assay is a quantitative PCR assay (FAM-MGB dual-labelled probe) with RNaseP (VIC-TAMRA dual-labelled probe) as the reference assay, performed as a duplex reaction. The test assay, the reference assay, the DNA sample and TaqMan® Master Mix were combined according to manufacturer’s instruction and run on an Applied Biosystems 7500 Real-Time System. All samples were tested in triplicate using 20 ng of DNA per reaction. Analyses were performed on a plate by plate basis, with analyses settings: Automatic baseline, and manual C_T_ threshold of 0.2 (as recommended by the manufacturer). The CN for each sample was assigned using CopyCaller™ software version 2.0 (Applied Biosystems), which uses relative quantitative analysis. The relative CN was determined on the basis of the comparative ΔΔC_T_ method with a normal control DNA as the calibrator on each plate (selected from the SNP array results to have 2CN). We excluded wells with VIC C_T_ greater than 32 and a zero copy ΔC_T_ threshold value of 4.0 (as recommended by the manufacturer). A confidence level of 95% and z-score value of <1.75 was applied to call the CNVs.

## Results

### Samples and genotyping

All samples retained for analyses showed a genome-wide call rate >99.5% for the Illumina 610-Quad array. Four samples were excluded from the analyses due to low call rates (<99.5%) in GenomeStudio, high quality score (>0.13) in Nexus and/or noisy log R ratio plots indicating poor genotyping efficiency. All four samples were LS cases (mutation positive for *MLH1* or *MSH2*).

### CNV association analyses

**Figure 1:**
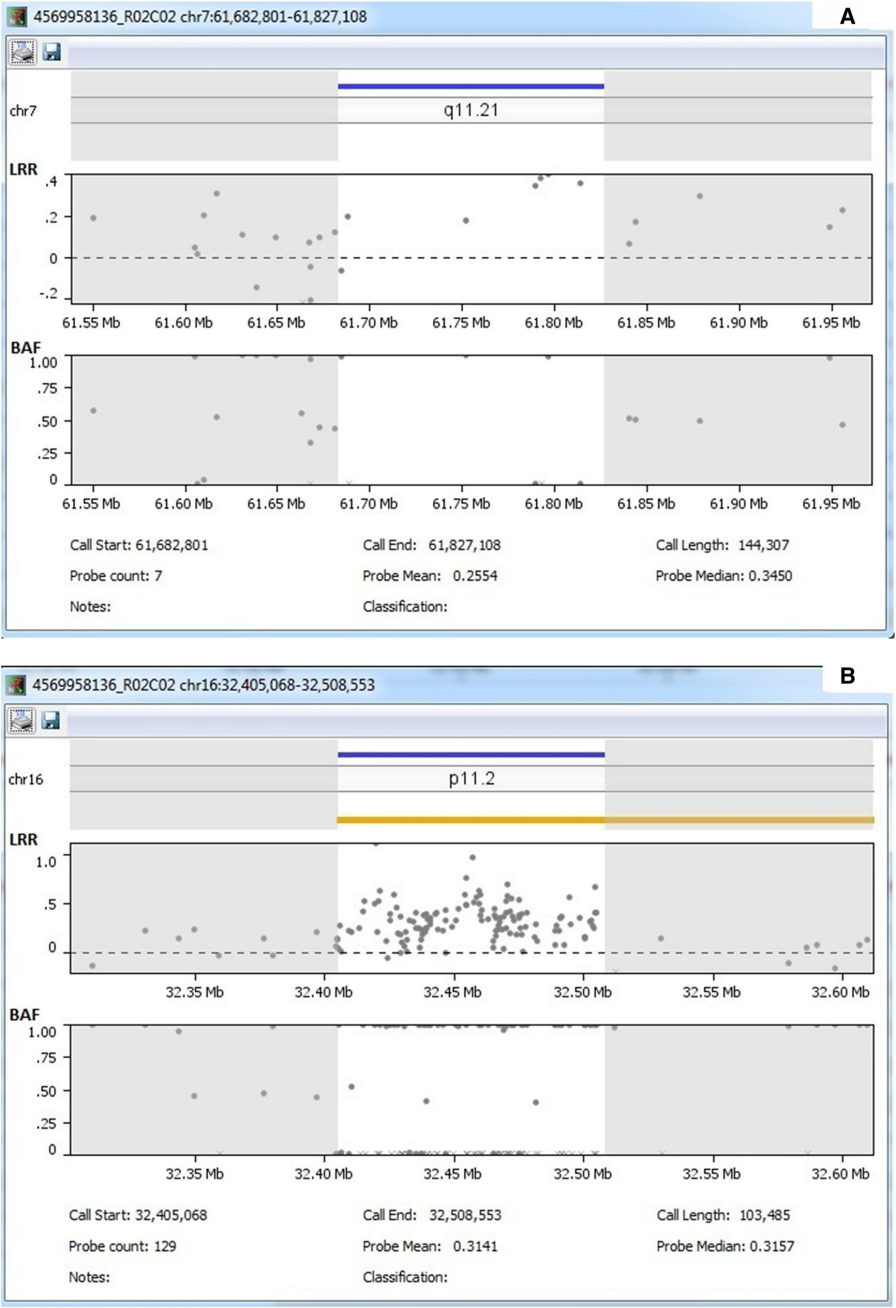
Displays log-R-ratio (LRR)/B-allele frequency (BAF) traces of the two significant regions, in addition to call start, call end, call length, probe count and probe median for; A) CN gain on chromosome 7q11.21 and B) CN Gain on chromosome 16p11.2.

Nexus software was used to conduct statistical comparisons of the frequency of each CNV event between cases and controls, with a differential threshold of 25%. The analyses identified two CNV events (CN gains on chromosomes 7q11.21 and 16p11.2) with significant frequency differences between LS/HNPCC cases (n = 96) and healthy controls (n = 384), see Table 3. The CN gain on chromosome 7q11.21 spans 140 kb and contained 7 SNP probes (see Figure 1A). It was observed in 28% of the LS/HNPCC cases, while none of the controls displayed a gain in this region (Nexus; *p* = 4.93E-21 and QuantiSNP; *p* = 5.00E-06). The CN gain on chromosome 16p11.2 spanned 82 kb and contained 122 probes: 6 SNP probes and 116 cnv probes (see Figure 1B). It was observed in 46% of the LS/HNPCC cases, whereas the reverse copy number state was observed in the control group; CN loss in 23% of the controls (Nexus; *p* = 3.60E-20 and QuantiSNP; *p* < 1.00E-16). The two significant CNVs were both CN gains and located in regions containing no annotated genes, miRNA’s or CpG islands. For both CNVs, a higher frequency was observed in the LS/HNPCC cases compared to controls.

**Table 3:** Summary of the CN events were the frequency of the event was significantly different between cases and controls are listed in the table below

**Chromosome region**	**CN region**	**CN size**	**CN event**	**Frequency in cases**	**Frequency in controls**	**96 HNPCC/LS cases vs. 384 Controls**	
						**Nexus**	**QuantiSNP**
**7q11.21**	61,682,801-61,827,108	140 kb	CN Gain	28%	0%	***p*=4.93E-21**	***p*=5.00E-06**
**16p11.2**	32,411,929-32,504,942	82.0 kb	CN Gain	46%	CN Loss 23%	***p*=3.6E-20**	***p*<1.00E-16**

Nexus protocol 5 [53] was used to compare the sample groups. No genes, microRNA or CPG islands are present in any of the CN regions listed in the table.

### Overall Copy Number Variation burden

Data outputs from Nexus and QuantiSNP were used when calculating the overall CNV burden (total CNV length, average CNV length and number of observed CNV events) in each group of samples. Comparison of total and average CNV length revealed a greater total genomic CNV length (Nexus; *p* = 0.0006 and QuantiSNP; *p* = 0.0001) and a greater average CNV length (Nexus; *p* = 0.0044 and QuantiSNP; *p ≤* 0.0001) in the cases compared to the controls, see Table 4. A higher number of CNV events was also observed in LS/HNPCC cases compared to controls utilising the Nexus program (*p ≤* 0.0001) but not with QuantiSNP (*p* = 0.8981), see Table 4. The frequency of called events differed substantially between the methods, with Nexus calling a mean 233 CNVs in cases and a mean 50 CNVs in controls, while the equivalent frequencies for QuantiSNP were a mean 16 CNVs each called in cases and controls.

**Table 4:** CNV burden

**Sample group**	**Total CNV length**		**Average CNV length of CNVs**		**Number of CNV’s**	
**Software**	**Nexus**	**QuantiSNP**	**Nexus**	**QuantiSNP**	**Nexus**	**QuantiSNP**
**HNPCC vs. Controls**	***p*** **= 0.0006**	***p*** **= 0.0001**	***p*** **= 0.0044**	***p*** **≤ 0.0001**	***p*** **≤ 0.0001**	*p* = 0.8981
**96 LS/HNPCC probands**						
Mean (95% CI)	31.2 Mb (15.4 – 46.9 Mb)	1.4 Mb (1.2-1.7 Mb)	82.5 kb (68.5-96.5 kb)	87.8 kb (75.0-100.6 kb)	233 (186–281)	16 (14–17)
**384 Healthy controls**						
	3.1 Mb (2.7-3.6 Mb )	900 kb (847–953 Kb)	61.7 kb (59,6-63,7 kb)	56.9 kb (53.7-60.2 kb)	50 (46–54)	16 (15–16)
Mean (95% CI)						
**60 MMR + vs. 36 MMR-**	*p* = 0.8630	*p* = 0.6355	*p* = 0.6302	***p*** **= 0.0069**	*p* = 0.0509	***p*** **= 0.0021**
**60 MMR + LS probands**						
Mean (95% CI)	30.0 Mb (11.7-48.3 Mb)	1.4 Mb (1.0-1.7 Mb)	85.3 kb (68.7-101.8 kb)	72.4 kb (61.0-83.4 kb)	199 (135–264)	17 (15–19)
**36 MMR- HNPCC probands**						
Mean (95% CI)	33.0 Mb (2.9-63.1 Mb)	1.5 Mb (1.1-1.9 Mb)	77.9 kb (51.6-104.1 kb)	11.3 kb (86.2-140.6 kb)	290 (224–356)	13 (11–14)

Nexus and QuantiSNP output analysis of overall CNV burden; total length of CNV’s, average length of CNV’s and number of CNV’s in Lynch syndrome/HNPCC patients (n = 96) vs. Controls (n = 384) and between MMR + individuals (n = 60) vs. MMR- individuals.

We also compared the total and average CNV length between mutation positive LS (MMR+) cases (n = 60) and mutation negative HNPCC (MMR-) cases (n = 36). No difference was observed in the total length of CNV’s (Nexus; p = 0.8630 and QuantiSNP; *p* = 0.6355), but a significantly greater average length was observed in the MMR positive group for QuantiSNP (*p* = 0.0069) but not for Nexus (*p* = 0.6302). When comparing the total number of CNV events between the two groups Nexus suggested a trend towards significantly more events for the MMR negative group (*p* = 0.0509), but the converse was observed in QuantiSNP (*p* = 0.0021) and to a much lesser degree; likely reflected by the marked difference in event frequencies called by the two methods. See Table 4.

### Unique Copy Number Variation burden

We also conducted analyses of CNV burden that included only unique/rare CNV events (defined as CNV events showing 0% overlap with previously reported CNV’s). These analyses were restricted to Nexus output, due to the difficulty of defining unique CNVs using QuantiSNP. The total length, average length and number of CNVs were much higher in LS/HNPCC samples compared to controls, see Table 5. The mean total length in LS/HNPCC patients was 2.9 Mb greater than in the controls (*p ≤* 0.0001), the average CNV length was 9 kb greater than in controls (*p ≤* 0.0001), and there were 70 more CNVs in LS/HNPCC patients (mean 84) compared to controls (mean 14), *p ≤* 0.0001. No significant difference was observed in the total and average length between MMR + LS cases vs. MMR- HNPCC cases but the number of CNV events was higher in MMR- cases (mean 117) compared to MMR + cases (mean 64), p = 0.0007.

**Table 5:** Unique CNV burden

**Sample group**	**Total CNV length**	**Average length of CNVs**	**Number of CNV’s**
**96 LS/HNPCC vs. 384 Controls**	***p*** **≤ 0.0001**	***p*** **≤ 0.0001**	***p*** **≤ 0.0001**
**96 LS/HNPCC proband**			
Mean (95% CI)	3.3 Mb (2.5-4.1 Mb)	33.3 kb (31.2-35.4 kb)	84 (68–100)
**384 Controls**			
Mean (95% CI)	392.3 kb (323.2-461.3 kb)	23.4 kb (22.3-24.4 kb)	14 (12–16)
**60 MMR + vs. 36 MMR- LS/HNPCC**	*p* = 0.0597	*p* = 0.9115	***p*** **= 0.0007**
**60 MMR + LS/HNPCC probands**			
Mean (95% CI)	2.8 Mb (1.7-3.8 Mb)	33.2 kb (30.2-36.2 kb)	64 (44–83)
**36 MMR- LS/HNPCC probands**			
Mean (95% CI)	4.3 Mb (3.1-5.5 Mb)	33.4 kb (30.9-36.0 kb)	117 (93–140)

Nexus output analysis of unique CNV burden; total length of CNV’s, average length of CNV’s and number of CNV’s in HNPCC patients (n = 96) vs. Controls (n = 384) and between MMR + individuals (n = 60) vs. MMR- individuals (n = 36).

### Validation of CN gains on chromosome 7q11.21 and 16p11.2

The concentrations of all the DNA samples were normalised and twice confirmed using Epoch™ Spectrophotometer System (Take3™ Multi-Volume Plate). DNA quality was considered to be good (sample purity: OD_260/280_ = 1.8-2.0). Neither of the TaqMan® Custom CN Assays validated the array results. The assay designed for the CN gain on chromosome 7q11.21 produced average confidence values across the samples of 98% (when confidence values of <50 and >99% was set to 49 and 99% respectively (standard deviation = 7%). Only two of the expected twenty-two (9%) cases displayed a CN of 3 (3CN), one with a confidence level of <50% and the other >99%. Two samples indicated a 3CN (confidence level of 95 and 99%) when initial array results suggested a normal CN state. Repeating the assay produced CN calls that were inconsistent with those of the initial run. Attempting to validate the CN gain on chromosome 16p11.2 produced low confidence values across the samples (average confidence values is 80%, standard deviation = 24%), and only 12 of the expected 36 (33%) samples demonstrated a CN > 2 (confidence values ranging from <50 – 89%). Three samples indicated a CN of 3 (all with a confidence level of <50%) when initial array results suggested a normal CN state.

### Dataset re-analysed on Nexus v.6.1

Nexus v.6.1, a specific version with linear correction, became available in the latter stages of this project. The dataset was re-analysed with the same analysis settings as before. Nexus v.6.1 allows linear correction to be applied; the bias values in the columns of the correction file (e.g. GC%, PCR fragment GC%, fragment length) are used to create a linear model whose parameters are estimated using the least squares method. The estimate is then subtracted from the probe Log_2_Ratio to obtain the corrected probe values. Linear correction was applied to our dataset, resulting in 9% of the probes being discarded. Interestingly, the CN gain on chromosome 16p11.2 was not observed in this analysis and after further investigation we saw that all of the cnv probes in this region were discarded. Only the SNP probes were retained and they did not display a CN gain. The CN gain on chromosome 7q11.21 identified by Nexus v.5.0 was still evident, but in fewer samples than before and the region no longer display a significant frequency difference between cases and controls.

### Comparison of the most significant results

Due to the inconsistency in the results between Nexus and QuantiSNP software’s we decided to compare the most significant results in more detail by repeating the association analysis between cases and controls with a differential threshold of 10%, see Table 6. We procured a table of CNVs showing significant frequency differences at p < 1x10E-10 from the Nexus output (17 CNVs) and compared it to QuantiSNP results. In 8 out of 13 CNV regions QuantiSNP called longer regions than Nexus and in 4 CNV regions from Nexus output no overlapping CNVs was detected in the QuantiSNP results. Frequency differences in cases and controls can be observed between the software’s, see Table 6.

**Table 6:** A table of the most significant results from the repeated association test between cases and controls including details of CNV region, cytoband location, event, region length, frequency in cases and controls, p-value and % of CNV overlap from Nexus output and CNV region, number of SNPs in segment, frequency in cases and controls, and p-value for QuantiSNP results

**Nexus**								**QuantiSNP**				
**Region**	**Cytoband location**	**Event**	**Region length**	**Freq. in cases (%)**	**Freq. in controls (%)**	**p-value**	% of CNV Overlap*	**Region**	**# SNPs in segment**	**Freq. in cases (%)**	**Freq. in controls (%)**	**p-value**
chr1:1,082,510-1,109,835	p36.33	CN Loss	27325	14.6	0.0	7.33E-11	100	chr1:1,064,487-1,096,336	8	0.0	0.3	0.62
chr1:192,838,687-193,008,078	q31.3	CN Gain	169391	15.6	0.0	1.29E-11	8	No overlapping CNV	─	0.0	0.0	NA
chr3:90,524,766-90,576,572	p11.1	CN Gain	51806	14.6	0.0	7.33E-11	100	chr3:90,421,209-90,576,572	19	16.7	0.0	4.06E-16
chr5:104,661,153-104,676,508	q21.3	CN Gain	15355	14.6	0.0	7.33E-11	100	chr5:104,667,691-104,675,112	5	1.0	0.0	0.05
chr6:31,945,137-31,947,946	p21.32	CN Loss	2809	14.6	0.0	7.33E-11	0	No overlapping CNV	─	0.0	0.0	NA
chr6:62,208,962-62,262,670	q11.1	CN Gain	53708	17.7	0.5	4.66E-11	100	chr6:62,176,064-62,260,258	11	15.6	0.0	3.55E-15
chr7:61,644,365-62,087,478	q11.21	CN Gain	443113	28.1	0.0	4.93E-21	100	chr7:61,667,556-61,990,710	18	7.3	0.0	9.79E-08
chr8:145,462,650-145,641,721	q24.3	CN Loss	179071	17.7	0.0	3.87E-13	100	No overlapping CNV	─	0.0	0.0	NA
chr9:138,620,572-138,764,838	q34.3	CN Loss	144266	15.6	0.0	1.29E-11	100	No overlapping CNV	─	0.0	0.0	NA
chr9:9,793,206-9,814,023	p23	CN Gain	20817	15.6	0.0	1.29E-11	100	chr9:9,778,666-9,809,028	21	1.0	0.0	0.05
chr11:50,339,475-50,370,127	p11.12	CN Gain	30652	21.9	0.0	3.08E-16	100	chr11:50,654,023-50,961,054	6	5.2	0.0	6.94E-06
chr11:54,468,566-54,554,469	q11	CN Gain	85903	20.8	0.3	3.30E-14	100	chr11:54,468,566-54,533,370	19	13.5	0.3	4.62E-12
chr12:36,616,479-36,650,608	q12	CN Gain	34129	19.8	0.5	1.66E-12	100	chr12:36,301,572-36,667,312	23	9.4	0.3	2.24E-08
chr14:104,706,668-104,721,437	q32.33	CN Loss	14769	14.6	0.0	7.33E-11	100	chr14:104,688,087-104,717,224	10	1.0	0.0	0.05
chr16:32,405,679-32,504,942	p11.2	CN Gain	99263	45.8	4.2	1.98E-22	100	chr16:32,443,063-32,460,991	23	45.8	20.1	1.96E-07
chr18:15,069,391-15,093,669	p11.21	CN Loss	24278	14.6	0.0	7.33E-11	100	chr18:15,045,092-15,219,051	20	8.3	0.0	1.17E-08
chr19:32,445,280-32,903,861	q12	CN Gain	458581	24.0	0.3	1.65E-16	100	chr19:32,520,504-32,810,457	28	11.5	0.3	3.27E-10

* Defined by Nexus as being reported in public repositories as being normal polymorphic in the "normal population".

Footnote:

The table shows CNVs showing significant frequency differences (at P < 1x10E-10) between HNPCC cases and controls in the analysis using Nexus.

For QuantiSNP results, Fisher's exact test was used for simple comparisons of CNV frequencies between cases and control (different p-values from Table 3).

## Discussion

The potential discovery of CNVs associated with HNPCC/LS would represent a significant advance in the search for genetic loci associated with disease expression. In the current study, using Illumina SNP arrays we identified two CN gains (7q11.21 and 16p11.2) with significant frequency differences between cases and controls. The CN gain on chromosome 16p11.2 could not be validated with a CN assay and was not evident when the dataset was re-analysed on a newer version of Nexus software (v6.1) and is therefore considered to be a false positive observation from our primary analyses. False CNV calling may be caused by intensity fluctuations on SNP arrays, which have been shown to occur as a result of the GC content of probed sequences, the position of the SNP in the probe and algorithms used to analyse array signals [55]. It is likely that the detected CN gain at chromosome 16p is an artefact of some or all of these phenomena, and this is supported by the exclusion of these probes when Nexus 6.1 linear correction was applied.

The CN gain on chromosome 7q11.21 could not be validated by TaqMan assay but is still evident when re-analysed on Nexus v6.1 (18% of cases still have CN gain, while none of the controls display a CN gain in the same region). Unlike TaqMan® Pre-Designed and Custom Plus assays, the Custom assay design used for these validations does not go through genome quality checks as the others and is designed on a masked sequence provided by the customer. The Custom assay for the CN gain on chromosome 7q11.21 demonstrated a weakness in its reproducibility (low confidence scores and inconsistent calls when repeated), which may or may not be a result of DNA sequence-specific complications. These results are evidence of loci specific, elevated rates of false detection for both platforms used, and since all sample concentrations were equilibrated and pipetting between plates was consistent, a technical cause of this inconsistency could not be identified. Due to the difficulty designing a CN assay in the two regions, only one CN assay was designed in each region. This is a possible limitation in the attempt to validate the results, as two assays within the segment and one assay outside the segment (as negative control) would have been optimal.

Another method that was available to validate the CN gains at 7q11.21 and 16p11.2 was Affymetrix 2.7 M array results from another project (unpublished data) that included 30 of our cases. Neither of the two regions is covered by this array – and when further investigated, Affymetrix informed that the probe performance over these regions was not optimal. The Applied Biosystems Custom Plus assay design service was unable to design suitable assays for these regions, perhaps indicating a similarly reduced capacity for optimal data acquisition and may be reflective of the poor data obtained.

The CN gain on chromosome 7 is located in a chromosomal region where there are no annotated genes/miRNA/CpG islands, but the CN gain is downstream of a CpG island (CpG: 139) and upstream of the gene LOC643955 (function unknown). The importance of these intergenic regions is poorly understood but they may be involved in regulating the expression of up- or down-stream genomic regions [56] or be in linkage disequilibrium with disease associated regions. CNVs in the region have previously been reported in control populations [57]. Chromosome 7q11-21 has previously been associated with cancer [58,59] and interestingly, both regions identified in the current study (7q11.21 and 16p11.2) have been found as CN gains in small bowel adenocarcinomas [60], which raises questions whether this is evidence in favour of the findings of the current study, or calls into question the stringency of the analysis which reported it.

It has been suggested that the overall CNV burden creates a differing sensitised background during development, leading to different thresholds of disease [61]. In the current study we observed that HNPCC/LS cases have a greater overall CNV burden and unique/rare CNV burden compared to controls. This is consistent with previous reports for other complex genetic disorders. For example, individuals with schizophrenia have a greater genomic burden of structural variation compared to controls [62] and rare CNVs have been observed in schizophrenia patients but not controls, supporting a disease model incorporating the effects of multiple, rare, highly penetrant variants [63]. Few studies have investigated germline CNVs and cancer risk, but the total number of germline CNVs have been found to be higher in patients with Li-Fraumeni syndrome compared to controls [64]. A large CNV burden has also been positively correlated with the severity of childhood disabilities [24]. In the current study, the high overall CNV burden in HNPCC/LS patients could be due to their MMR deficiency arising from mutations in MMR genes, supporting the idea that deficiency of MMR occurs first and the adenoma evolves from the MMR-deficient cell [65]. Therefore we tested the overall difference in the CNV burden between MMR + LS patients and MMR- HNPCC patients. The total and average CNV length was not different between the two groups but the number of CNV events was. Interestingly, Nexus Software analysis suggested that MMR- HNPCC cases had a greater unique/rare CNV burden than MMR + probands, which could be an indication of a deficient DNA repair in these patients despite the negative mutation screen in MMR genes known to be associated with the disease. Because our clinical cohort represents a highly ascertained population that underwent CNV analyses as a result of a clinical/molecular diagnosis of HNPCC/LS, the subjects are possibly enriched for rare CNVs. However, we only cautiously suggest this interpretation, due to the described challenges with validating Nexus results.

We took a rigorous and conservative analytical approach to maximise CNV call reliability by calculating NSR, setting the number of probes to a minimum of 5 and using two different algorithms to identify significant CNV differences between cases and controls. Utilising more than one algorithm in CNV calling have been applied in several studies to improve the rates of reproducibility and positive prediction [19,66-69], however it invariably demonstrates an increase in the overall false positive rate. Accordingly, we sought to control our positive prediction rate by considering only those regions that satisfied dual algorithm detection at respective significance thresholds as qualifiers of association with LS/HNPCC. Nevertheless, our findings should be interpreted with caution as we can see considerable differences between the CNV frequencies detected in cases/controls in the association analysis, the total length, average length and the number of CNVs called between the two software programs used for analysis. Reassuringly, the discrepancies we observed are consistent with the results of other recent studies that have attempted to use convergence across multiple algorithms to identify valid CNV calls [67-69]. The source of these discrepancies is due to the differing sensitivities of algorithms to the inherent variations in relative fluorescence between co-assayed genomic loci on SNP arrays.

To compare the algorithms used in the current study, Nexus uses a proprietary CBS based algorithm to divide chromosomal data into segments whose median LRR values are significantly different from adjacent segments. CNVs are defined using numerous one-size-fits-all user defined thresholds (see methods) and may therefore be susceptible to CNV call reliability fluctuations according to data quality. The Nexus algorithm only considers single samples for CNV calling and does not draw on collective data for greater call confidence. Conversely, QuantiSNP uses a HMM where aberrations are defined as excursions from the null state that satisfy multiple parameters learnt from the input data and confidence is heightened if the aberrations are detected in multiple samples. Additionally, there are only two user defined thresholds (the characteristic length parameter (2 MB default) and Log-Bayes Factor), which serve to reduce the false positive error rate at differing stages of the analysis [52].

The challenge seems to be a combination of the inherent inaccuracy of measuring signal intensity using genotyping data from SNP arrays and systematic differences between statistical algorithms. Accordingly, the observed higher Type I error rate by Nexus in the current study may be due to the lack of control for false positives, the rigidity of its user-defined thresholds which do not adapt in line with data quality and a lack of confidence testing of aberrations (that is, the only significance testing is applied at the segmentation stage, not when the LRR ± cut-offs indicate CN gain or loss). In recent comparisons [34,70], both programs utilised in the current study have performed well compared to other algorithms and we used settings consistent with those previously reported. The recent study by Kim *et al.*[69] suggests that convergent CNV calls across at least three algorithms should be obtained before undertaking association analysis as only ~10% of CNVs called using two algorithms were verified by a third. Such low convergence likely reflects a combination of type I and type II error across the discovery and validation analysis. Kim *et al.*[69] do however show that validity can be increased by increasing the CNV filtering criterion to require the inclusion of at least 7 probes, suggesting that better validity may have resulted from applying a third algorithm and requiring called CNVs to contain at least 7 consecutive probes.

New software for analysing CNVs is being rapidly developed [34,70] and as no gold standard has yet been established, CNV analyses remains challenging and the results difficult to interpret. Other possible limitations of our study are the control population, the modest sample size and the potential for false negative results due to strict analytical parameters. The controls were healthy individuals at the time of sampling but may develop cancer in the future, which would be expected to reduce power of our analyses. However, all controls were aged >55 years, which reduces the potential impact of misclassification bias.

The genomic region on chromosome 7q11.21 requires further investigation to prove the association with the investigated disease and should not be dismissed due to its location in an intergenic region. The HNPCC/LS cases have a greater burden of CNV across their genomes compared to controls which is supporting the notion of higher genomic instability in these patients due to an inadequate DNA repair process. The technology is improving rapidly, but until next-generation sequencing is available and widely used in clinical diagnostic testing, inspecting the overall CNV burden in individuals with a clinical diagnosis of HNPCC/LS could become a rapid and cost-efficient screening method for identifying families for genetic testing. Future research should explore the identified candidate locus on chromosome 7q11.21 further as well as consider whether high CN at this locus increases the risk of disease development in the context of HNPCC/LS families.

## Conclusion

In conclusion, we have identified a greater CNV burden in HNPCC/LS cases compared to controls supporting the notion of higher genomic instability in these patients due to an inadequate DNA repair process. One intergenic locus on chromosome 7q11.21 is possibly associated with disease risk in patients diagnosed with HNPCC/LS and should therefore not be dismissed as a false positive without further investigation. The results from this study highlight the complexities of fluorescent based CNV analyses; the inefficiency of both CNV detection methods to reproducibly detect observed CNVs demonstrates the need for sequence data to be considered alongside intensity data to avoid false positive results.

## Competing interests

The authors declare that they have no competing interests.

## Authors’ contributions

BTP conceived the study, participated in its design and carried out the molecular genetic studies, analysis and drafted the manuscript. EGH and TJE participated in the design and analysis of the study. MM and JA: Control collection and material support. DMG and ALM participated in the completion of the study. CM and AS: Patient collection and material support. RJS participated in study design and coordination. All authors read and approved the final manuscript.

## Pre-publication history

The pre-publication history for this paper can be accessed here:

http://www.biomedcentral.com/1755-8794/6/10/prepub
